# Human Apolipoprotein A-I Natural Variants: Molecular Mechanisms Underlying Amyloidogenic Propensity

**DOI:** 10.1371/journal.pone.0043755

**Published:** 2012-08-28

**Authors:** Nahuel A. Ramella, Guillermo R. Schinella, Sergio T. Ferreira, Eduardo D. Prieto, María E. Vela, José Luis Ríos, M. Alejandra Tricerri, Omar J. Rimoldi

**Affiliations:** 1 Instituto de Investigaciones Bioquímicas de La Plata (INIBIOLP), CONICET, La Plata, Buenos Aires, Argentina; 2 Facultad de Ciencias Médicas, Universidad Nacional de La Plata, La Plata, Buenos Aires, Argentina; 3 Cátedra de Farmacología Básica, Facultad de Ciencias Médicas, Universidad Nacional de La Plata, La Plata, Buenos Aires, Argentina; 4 Comisión de Investigaciones Científicas, Pcia de Buenos Aires, La Plata, Argentina; 5 Program in Biochemistry and Cellular Biophysics, Institute of Medical Biochemistry, Federal University of Rio de Janeiro, Rio de Janeiro, Rio de Janeiro, Brazil; 6 Instituto de Investigaciones Fisicoquímicas Teóricas y Aplicadas (INIFTA), Universidad Nacional de La Plata-CONICET, La Plata, Argentina; 7 Departament de Farmacologia, Facultat de Farmacia, Universitat de Valencia, Valencia, España; University of South Florida College of Medicine, United States of America

## Abstract

Human apolipoprotein A-I (apoA-I)-derived amyloidosis can present with either wild-type (Wt) protein deposits in atherosclerotic plaques or as a hereditary form in which apoA-I variants deposit causing multiple organ failure. More than 15 single amino acid replacement amyloidogenic apoA-I variants have been described, but the molecular mechanisms involved in amyloid-associated pathology remain largely unknown. Here, we have investigated by fluorescence and biochemical approaches the stabilities and propensities to aggregate of two disease-associated apoA-I variants, apoA-IGly26Arg, associated with polyneuropathy and kidney dysfunction, and apoA-ILys107-0, implicated in amyloidosis in severe atherosclerosis. Results showed that both variants share common structural properties including decreased stability compared to Wt apoA-I and a more flexible structure that gives rise to formation of partially folded states. Interestingly, however, distinct features appear to determine their pathogenic mechanisms. ApoA-ILys107-0 has an increased propensity to aggregate at physiological pH and in a pro-inflammatory microenvironment than Wt apoA-I, whereas apoA-IGly26Arg elicited macrophage activation, thus stimulating local chronic inflammation. Our results strongly suggest that some natural mutations in apoA-I variants elicit protein tendency to aggregate, but in addition the specific interaction of different variants with macrophages may contribute to cellular stress and toxicity in hereditary amyloidosis.

## Introduction

Certain proteins require a high degree of conformational flexibility in order to fulfill their biological functions. Those proteins, however, are exposed to the risk of a shift in equilibrium between the folded, native structure and a conformation prone to undergo self-aggregation. Amyloidoses are characterized by aggregation and deposition of insoluble protein fibrils, with concomitant destruction of normal tissue functionality. Despite the fact that such protein deposits are morphologically similar, more than 25 unrelated proteins have been found to be associated to amyloid diseases [1]–[3]. Different mechanisms appear to be involved in the conversion of a protein from a soluble, native state into an aggregated, misfolded form, including an intrinsic propensity to assume a pathological conformation, which becomes evident with aging [4], proteolytic processing of a precursor protein, as is the case of the Aβ peptide in Alzheimer’s disease [5] or replacement of a single amino acid residue, as described for different hereditary amyloidoses [4], [6]. In addition, other factors including local increases in protein concentration [7] and/or changes in physicochemical properties of the medium [8] have been shown to affect amyloid aggregation.

Human apolipoprotein A-I (apoA-I) is the major protein component of high density lipoproteins (HDL) serving as transporters for excess cellular cholesterol through the plasma compartment to the liver. Even though many steps are involved in this process, it has been suggested that the efficiency of apoA-I *in vivo* is a direct function of its ability to dissociate from HDL particles and remain stable as lipid-poor forms that can be rapidly lipidated [9].

Hereditary apoA-I amyloidosis is a rare, late-onset, autosomal dominant condition characterized by systemic deposition of amyloid in tissues, the major clinical problems being related to renal [10], [11], hepatic [12], and cardiac involvement [13]. Other tissues and organs less frequently involved include the skin, testes, larynx and peripheral nerves [14], [15]. Interestingly, amyloid deposits of apoA-I in the aortic intima are often associated with atherosclerotic plaques, notably in patients carrying the apoA-ILys107-0 deletion mutant [16]. More than 50 natural variants of apoA-I have been described, and about one third of them is associated with familial amyloidosis [6]. As the economy improves in countries undergoing economic development, it seems likely that genetic studies will identify additional protein variants associated with this pathology. The reasons why each particular mutation induces apoA-I aggregation and deposition are still unclear. For example, some pro-amyloidogenic mutations involve replacement of neutral residues (Gly26, Trp50, Leu60, Leu178) by cationic amino acids, inducing a change in net charge of the protein [17]–[19]. However, similar mutations in other domains of the protein do not favor formation of insoluble aggregates [20], [21]. Moreover, while mutations that do not involve gain of positive charge induce amyloidosis [13], a variant in which two positive charges are gained by the protein (Glu110Lys) has been shown to be innocuous in terms of amyloid pathology [22].

In this study, we set out to investigate features involved in induction of amyloid aggregation from two natural single point mutants of apoA-I: the Iowa variant, in which a glycine amino acid is replaced by an arginine residue at position 26 (apoA-IGly26Arg), and the Helsinky variant, a deletion mutant lacking a lysine residue at position 107 (apoA-ILys107-0). These two variants are found in amyloid lesions and both were shown to be rapidly catabolized compared with normal apoA-I, accounting, at least in part, for low levels of HDL in patients carrying these mutations [23], [24]. Nevertheless, the organs affected in each case are different, suggesting that different structural features or different susceptibilities to micro environmental factors could determine the pathogenicity of these and other apoA-I mutants. Results presented here indicate that different conformational stabilities and ability to trigger pro-inflammatory responses are related to specific disease phenotypes associated with each variant.

## Materials and Methods

### Materials

Guanidine hydrochloride (GndHCl), thioflavin T (ThT), matrix metalloproteinase-12 (MMP-12, Catalytic Domain), 12-O-tetradecanoylphorbol-13-acetate (TPA), lipopolysaccharide (LPS), polymyxin B sulfate, 3-[4,5-dimethylthiazol-2-yl]-2,5-diphenyl tetrazolium bromide (MTT) and Hystopaque were from Sigma-Aldrich (St Louis, MO). His-purifying resin was from Novagen (Darmstadt, Germany). 4,4′-dianilino-1,1′-binaphtyl-5,5′-disulfonic acid, dipotassium salt (bis-ANS) was purchased from Molecular Probes (Invitrogen, Carlsbad, CA). All other reagents were of the highest analytical grade available.

### Methods

#### Cloning, expression and purification of wild-type apoA-I

The cDNA for human apoA-I, kindly donated by Dr A. Jonas (University of Illinois at Urbana-Champaign, IL), was further modified to introduce an acid labile Asp–Pro peptide bond between amino acid residues 2 and 3 of apoA-I, which allowed specific chemical cleavage of an N-terminal His-Tag fusion peptide [8], [25]. This construct, inserted into a pET-30 plasmid (Novagen, Madison, WI), was used to express wild type apoA-I, used as a control throughout this study. In addition, this plasmid worked as a template for construction of the single point substitution mutant Gly26Arg by the Quickchange method (Stratagene, La Jolla, CA). The deletion mutant Lys107-0 was obtained from a plasmid used previously [26] by further introduction of the acid-labile peptide bond [8]. Protein expression and purification were performed as described [8], resulting in a high yield of protein with a purity >95% (as determined by SDS-PAGE).

#### Protein structure under native conditions

ApoA-I variants were diluted to 0.1 mg/mL in citrate phosphate McIlvaines buffer, pH 7.4. Intrinsic fluorescence was measured at 25°C on an Olis upgraded SLM4800 spectrofluorometer (ISS Inc, Champaign, IL), with excitation at 295 nm. Solvent exposure of Trp residues was determined by fluorescence quenching induced by increasing concentrations of acrylamide as described previously [8], [27]. The recovered parameter K is the quenching constant.

Presence of exposed hydrophobic domains in the native structures of apoA-I variants was determined by binding of the fluorescence probe bis-ANS [28], [29]. Small aliquots of bis-ANS (from a concentrated stock solution in methanol) were added to apoA-I variants at 25°C and fluorescence emission spectra were acquired between 450–550 nm with excitation at 395 nm. Residual methanol concentration was kept to a minimum in order to avoid structural artifacts due to solvent effects.

#### Protein denaturation and stability

Chemical denaturation was performed by incubation of 0.1 mg/mL apoA-I in the presence of increasing concentrations of GndHCl at pH 7.4 and 25°C. Fluorescence emission spectra were acquired as described above [8]. The free energy of unfolding in the absence of denaturant (ΔG^0^) and the GndHCl concentration in which half of the protein is unfolded [GndHCl]_1/2_ were obtained from the shift in spectral center of mass of the fluorescence emission, assuming a two-state process as previously described [30], [31]. Alternatively, denaturation was monitored by incubating 0.1 mg/mL apoA-I variants with bis-ANS at a 1∶5 molar ratio (probe:protein) and measuring emission spectra after stepwise addition of GndHCl.

#### Morphology of apoA-I variants’ aggregates

Formation of protein aggregates from Wt apoA-I or disease-associated variants was monitored by Atomic Force Microscopy (AFM). Protein variants were incubated at 0.6 mg/mL for 24 h at 37°C, and spotted stepwise on freshly cleaved muscovite mica. The sample was blotted off with pure water to remove salts and dried under N_2_. All images were obtained in ambient conditions using a Multimode-Nanoscope V (Veeco, Santa Barbara, CA) operating in Tapping Mode with an etched silicon probe model Arrow-NCR-50 Nano World (cantilever resonance frequency: 258 kHz, force constant 42 N/m; tip radius 5–10 nm). Typical scan rates were 1 Hz-1.5 Hz.

#### Pro-inflammatory processing of apoA-I variants

We have previously reported that a pro-inflammatory microenvironment induces processing of Wt apoA-I into pro-amyloidogenic intermediate complexes [8]. In order to determine the relative susceptibilities of apoA-I variants to such processing, we incubated the proteins with activated neutrophils. Human polymorphonuclear neutrophils (PMNs) were isolated from venous blood of healthy volunteers, purified and resuspended as previously described [8]. ApoA-I variants (0.2 mg/mL) were added to 1×10^5^ cells in 500 µL and, after 5 min at 37°C, cells were stimulated with TPA (200 nM), followed by 1 h incubation. Reaction was stopped by spinning the cells at 1,000 ×*g* for 5 min. Proteins in the supernatant were then loaded onto a 12.5% SDS-PAGE gel and developed by Western blotting using a polyclonal antibody against apoA-I [32]. Aliquots of each apoA-I variant treated with PMNs under identical conditions were further incubated at 37°C for 24 h and used to analyze Thioflavin T (ThT) binding. ThT was added at a 1∶1 molar ratio and fluorescence intensities were measured on a Beckman Coulter DTX 880 Microplate Reader (Beckman, CA), using excitation and emission filters centered at 430 nm and 480 nm, respectively.

In a different experiment, apoA-I variants were incubated at 37°C for 3 h with matrix metalloproteinase-12 (MMP-12) (molar ratio 1∶3,000 MMP-12:apoA-I) and aliquots from the reaction mixture were analyzed by SDS-PAGE (as described above) for determination of released peptides. In additional aliquots, MMP-12 was inhibited by addition of EDTA (final concentration 5 mM), followed by 24 h incubation at 37°C to determine ThT binding as described above.

#### Assays with RAW 264.7 murine macrophages

RAW 264.7 murine macrophages (ECACC, Salisbury, UK) were maintained in DMEM supplemented with 10% fetal bovine serum (FBS), 100 U/mL penicillin, and 100 µg/mL streptomycin at 37°C in a humidified incubator containing 5% CO_2_. For all experiments, cells were subjected to no more than 20 passages.

#### a) 3-(4,5-dimethylthiazolyl)-2,5-diphenyl-tetrazolium bromide (MTT) cell viability assay

RAW 264.7 macrophages were seeded in 96-well plates at 6×10^4^ cells/well. After 24 h at 37°C medium was removed and DMEM supplemented with 0.5% FBS, 100 U/mL penicillin, and 100 µg/mL streptomycin was added in the absence or presence of 50 µg/mL polymyxin B. ApoA-I variants (1 µg/mL) or LPS were added and cellular redox activity, an indicator of cell viability, was quantified 24 h later by measuring the conversion of the tetrazolium salt MTT into its formazan product [33]. Briefly, after changing the medium, MTT was added to a final concentration of 0.5 mg/mL and cells were incubated for 20 min at 37°C. The medium was then removed and the formazan precipitate was solubilized in DMSO, and the absorbance measured at 490 nm on a microplate reader.

#### b) Effect on reactive oxigen species (ROS) production: DCFH-DA assay

Production of intracellular ROS from RAW 264.7 macrophage cells was evaluated by using the non-polar dye, dichlorofluorescein diacetate (DCFH-DA). The dye diffuses into cells, gets trapped by deacetylation, and in the presence of hydrogen peroxide becomes oxidized to yield 2′,7′-dichlorofluorescein (DCF). RAW 264.7 macrophages were plated as described above. After 24 h at 37°C, medium was removed and DMEM supplemented with 0.5% FBS, 100 U/mL penicillin, 100 µg/mL streptomycin and 50 µg/mL polymyxin B was added. ApoA-I (0.1 or 1.0 µg/mL) was added after one hour of incubation. LPS was used as a control. Six hours later, cells were washed with DMEM and incubated in presence of 20 µM DCFH-DA for 30 min at 37°C. After an extra wash with warm PBS, 100 µL passive lysis buffer (Promega, WI) were added to each well and fluorescence was measured on a microplate reader with excitation and emission filters centered at 485 and 525 nm, respectively.

#### c) Determination of tumor necrosis factor alpha (TNF-α) and interleukin-1β (IL-1β) production

RAW 264.7 macrophages were plated as described above. Cells were incubated in DMEM supplemented with 0.5% FBS, 100 U/mL penicillin, 100 µg/mL streptomycin and 50 µg/mL polymyxin B in the presence of apoA-I variants (0.1 or 1.0 µg/mL) at 37°C for 24 h. LPS was used as a control. The supernatants were then collected and assayed for TNF-α and IL-1β production using a specific enzyme immunoassay from eBioscience (San Diego, CA) used according to manufacturer's instructions.

#### Other analytical methods

Protein content was quantified by optical density in a Helios β spectrophotometer (Thermo Scientific, Waltham, MA), using an extinction coefficient of 32,430 M^−1^cm^−1^ at 280 nm.

Transmission electron microscopy was carried out on a JEOL-1200 EX microscope (School of Veterinary Medicine, National University of La Plata) operating at 100 kV. Samples (0.4 mg/mL) were incubated for 24 h at 37°C, applied onto Formvar-coated grids for 5 min and negatively stained with 2% uranyl acetate.

Unless otherwise stated, results are representative of three independent experiments. Results are means ± S.E of at least 3 samples. Statistically significant differences between experimental conditions were evaluated by ANOVA followed by Tukey’s test (*p*<0.05).

## Results

### Subtle Conformational Differences between Wt and Variant Forms of apoA-I in the Native State

**Figure 1:**
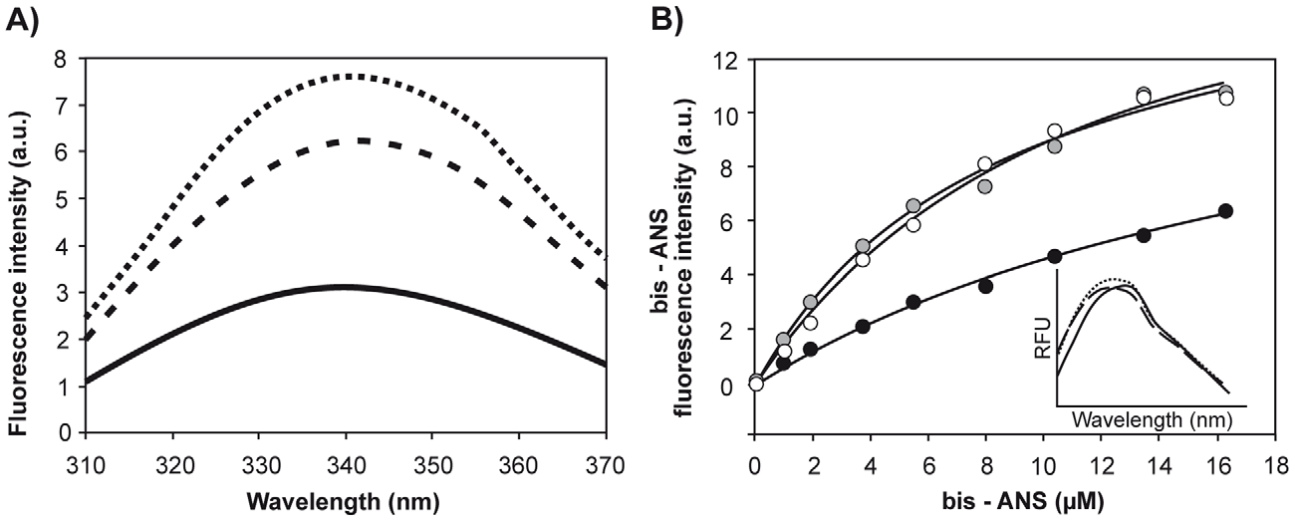
Characterization of apoA-I variants’ conformation. A) Intrinsic Trp fluorescence emission spectra of apoA-I variants. Proteins at a final concentration of 0.1 mg/mL in citrate phosphate Mc Ilvaines buffer pH 7.4. Excitation was set at 295 nm and emission was recorded between 310 and 370 nm. Continuous line represents Wt protein; Dashed and dotted lines are fluorescence spectra corresponding to Gly26Arg and Lys107-0 respectively. B) Fluorescence analysis of bis-ANS binding to apoA-I. ApoA-I variants, at a final concentration of 0.1 mg/mL were titrated with bis-ANS to a final concentration of 16 µM. The probe was excited at 360 nm, and emission registered as the Wavelength of Maximum Fluorescence for this probe. Dark circles represent the experimental data for Wt. Gray and white symbols correspond to Lys107-0 and Gly26Arg respectively. Inset: normalized fluorescence spectra of bis-ANS at a molar ratio 1∶1 probe to protein. Continuous line corresponds to Wt spectrum; Dashed and dotted lines correspond to Lys107-0 and Gly26Arg respectively.

We initially asked whether the two apoA-I variants investigated (Gly26Arg and Lys107-0 variants) exhibited any differences in native structure compared to Wt apoA-I. The intrinsic fluorescence emission of apoA-I corresponds to an average signal from four naturally occurring Trp residues (at positions 8, 50, 72 and 108 from the N-terminus), which are preserved in both amyloidogenic variants. The intrinsic fluorescence emission spectra of the two variants showed small but significant shifts of about 2 nm to longer wavelengths relative to Wt apoA-I (Table 1) and large increases in fluorescence quantum yields (Fig. 1A). Using site-directed mutagenesis and fluorescence energy transfer studies, Davidson et al [34] demonstrated that at least two of the Trp residues of apoA-I undergo fluorescence homotransfer, which leads to a decrease in quantum yield of the donor residues [34]. Because energy transfer is a distance-dependent process, the increase in intrinsic fluorescence quantum yield observed in the mutants could indicate a more relaxed structure, with the Trp residues on average more separated from each other that in the Wt protein.

**Table 1:** Spectral Properties and Stabilities of Wt apoA-I and the Gly26Arg and Lys107-0 mutants at 0.1 mg/mL, pH 7.4.

pH	Wt	Gly26Arg	Lys107-0
λ max Trp (nm)^(a)^	338.0±2.0	340.0±2.0	340.5±2.0
ΔG^o^ denat(kcal/mol)^(b)^	2.53±0.07	1.54±0.07	1.91±0.19
½ [GndHCl] (M)^(c)^	1.4±0.2	1.0±0.3	1.2±0.3
K (M^−1^)^(d)^	5.26±0.30	7.90±0.41	6.54±0.32

a Wavelength of maximum fluorescence of Trp residues, determined from the from the intrinsic fluorescence spectra as shown in Figure 1.

b and ^c^ Free energy change of unfolding and GndHCl concentration at which half of the protein is unfolded, respectively, calculated from equilibrium unfolding curves as described previously [8] and shown in Figure 2 (see “Methods").

d Stern-Volmer quenching constant (see “Methods").

In order to obtain additional information on the structural compactness of native apoA-I, we performed acrylamide quenching of the intrinsic fluorescence of both Wt and mutant forms of apoA-I. The quenching constant (K  = 5.26 M^−1^; Table 1) measured for Wt apoA-I is in good agreement with our previous report [8]. Interestingly, higher quenching constants were measured for both apoA-I variants (Table 1), indicating that the Trp residues in the variants are on average more exposed to the solvent than in Wt apoA-I.

We next compared bis-ANS binding to Wt and variant forms of apoA-I. Bis-ANS is an environment-sensitive fluorescent probe that binds to exposed hydrophobic surfaces in partially folded states more tightly than to native or fully unfolded proteins [28], [29], [31], [35], [36]. The quantum yield of bis-ANS is very low in aqueous solution, but increases sharply when bound to exposed hydrophobic areas in proteins. Titration of the native protein (3.6 µM) with bis-ANS showed non-cooperative binding (Fig. 1B). Significantly, bis-ANS fluorescence was higher in the presence of both mutants than in the presence of Wt apoA-I. Only minor spectral shifts were observed for bis-ANS bound to Wt or mutant forms of apoA-I (Fig. 1B, inset). Thus, the observed increase in fluorescence (Fig. 1B) is likely due to a higher number of bis-ANS molecules bound per protein [35]. Together, these results suggest that a single amino acid substitution (Gly26Arg) or deletion (Lys107-0) causes subtle changes in conformation of the N-terminal domain of apoA-I (where the Trp residues are located) that result in higher exposure of the Trp residues and of hydrophobic protein surface to the aqueous medium.

### Folding and Stability of Wt and Variant Forms of apoA-I

The equilibrium unfolding of Wt apoA-I by GndHCl has been well characterized [8], [37] and provides a useful tool to compare the stabilities of variants that are less known. As expected, the dependence of the shift in intrinsic fluorescence of Wt apoA-I on GndHCl concentration is described, at physiological pH (7.4), by a cooperative unfolding pattern that is well fit to a two-state model (black circles in Fig. 2A) [8], [38]. The calculated free energy of unfolding was 2.53 kcal/mol (Table 1), which suggests that native apoA-I exhibits a flexible structure likely resembling a molten globule state [39]. The denaturation profiles of both variants indicated lower stabilities, reflected in substantially lower [GndHCl]_1/2_ values (Table 1) and ΔG^0^ (1.91 and 1.54 kcal/mol for Gly26Arg and Lys107-0 variants, respectively). In addition, the less pronounced sigmoidal profiles of the curves suggests loss of folding cooperativity for both apoA-I variants (Fig. 2A).

**Figure 2:**
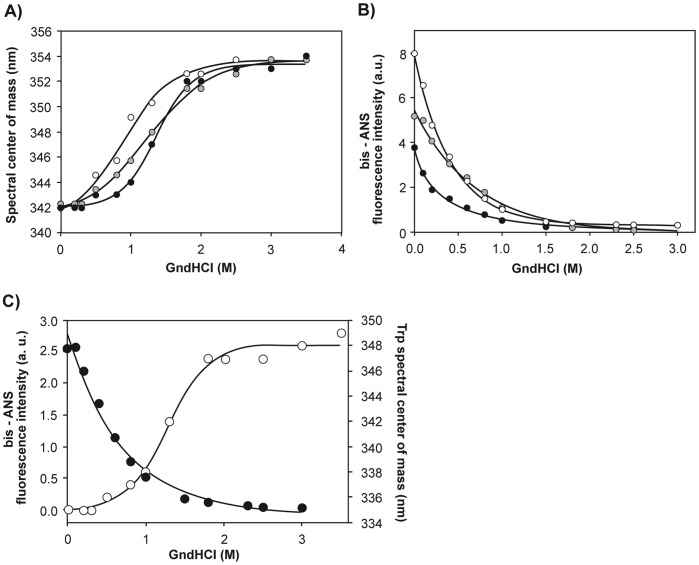
Chemical unfolding of apoA-I variants. Dark circles represent the experimental data for Wt. Gray and white symbols correspond to Lys107-0 and Gly26Arg respectively. A) Equilibrium unfolding of apoA-I variants as followed by intrinsic Trp fluorescence. Spectral centers of mass are plotted as a function of [GndHCl]. Final protein concentration was 0.1 mg/mL; excitation was set at 295 nm and emission recorded between 310 and 420 nm. Continuous lines are fits to the data, in the same order using a sigmoidal model. B) Dependence of bis-ANS fluorescence as a function of [GndHCl]. Proteins were diluted to 0.1 mg/mL and incubated with bis-ANS at a molar ration probe: protein 1∶5. GndHCl was added stepwise. Fluorescence was registered as the Wavelength of Maximum Fluorescence at each [GndHCl]. C) Overlap of GndHCl-mediated denaturation curves for Wt apoA-I as followed by Trp (panel A) and bis-ANS fluorescence (panel B).

The stabilities of Wt and variant forms of apoA-I were further investigated by measuring bis-ANS fluorescence in the presence of increasing concentrations of GndHCl. To this end, we incubated apoA-I with bis-ANS at a low molar ratio (protein:probe 5∶1), in order to avoid any shift in equilibrium from the native state due to excess binding of the probe [35]. As shown in Figure 2B, bis-ANS fluorescence decreased sharply in a continuous trend with increasing GndHCl concentrations, indicating that hydrophobic patches at the protein surface were maximally exposed in the native state and underwent progressive disorganization even at low GndHCl concentrations. This behavior was similar for Wt apoA-I and the two variants investigated, and showed an apparent [GndHCl]½ lower than 0.5 M GndHCl. Results demonstrated that the spatial arrangement of the proteins (as monitored by bis-ANS fluorescence) was disorganized at lower GndHCl concentrations than those needed to produce more global unfolding (revealed by the shift in intrinsic fluorescence emission). This fact is clear from the overlap of the GndHCl-dependent denaturation profiles revealed by both observables (Figs. 2A, B), and similar behavior was shared by Wt apoA-I and the two variants investigated. To illustrate this point, Figure 2C shows the overlap of denaturation curves monitored by bis-ANS and intrinsic fluorescence for Wt apoA-I. These data indicate the existence of different partially folded intermediates along the equilibrium unfolding of apoA-I.

### Morphology of the apoA-I Variants’ Aggregates

**Figure 3:**
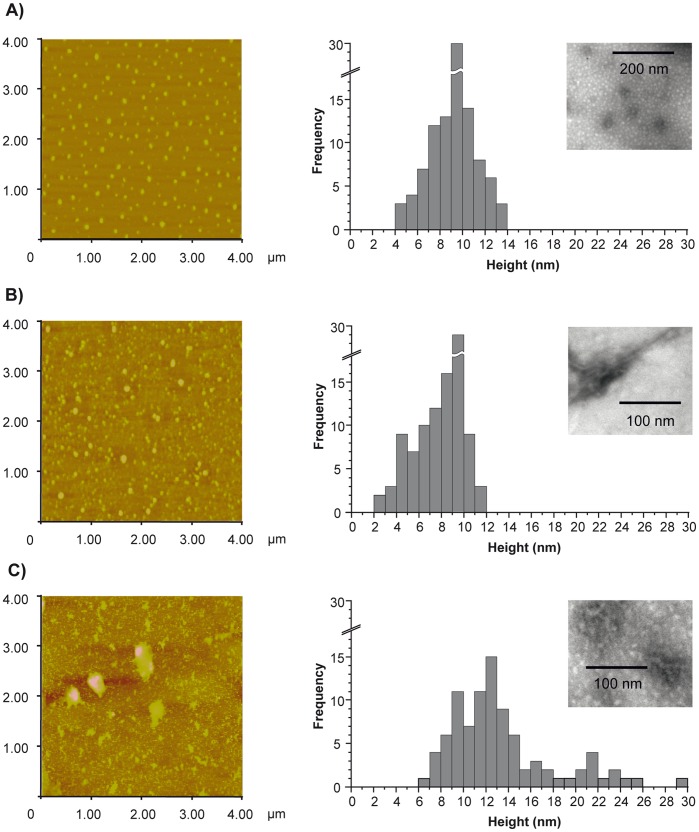
Morphology characterization of apoA-I mutants’ aggregates. Analysis of images observed under AFM. ApoA-I Wt (A), Gly26Arg (B) and Lys107-0 (C) respectively, were incubated for 24 h at 0.6 mg/mL and loaded onto mica. Small size oligomers covering the surface of the mica were predominant in each sample. The distribution of the oligomers’ height is shown as Histograms obtained from the measurement in the z-plane of 100 oligomers. Insets in the histograms’ plot represent isolated aggregates occasionally observed by negative stain under Transmission Electron Microscopy. Bars in each image show the scale used in each case.

In order to characterize the morphology of aggregates formed under our experimental conditions, samples were loaded onto a mica surface and observed under AFM. A pattern of small oligomers predominated for both Wt and variant forms of apoA-I. In order to estimate the dimensions of the oligomers, their height was measured using the Nanoscope 7.30 software. Figure 3 shows height distributions of aggregates formed from each mutant. Wt apoA-I preparations were characterized by oligomers exhibiting a homogeneous distribution centered at about 10 nm height on average (Fig 3A). A similar height distribution (slightly shifted to lower heights) was verified for the apoA-IGly26Arg variant (Fig 3B). In contrast, apoA-ILys107-0 aggregates were more heterogeneous with an average height around 10–14 nm and some of them exhibiting higher dimensions in the *z*-direction (Fig 3C). Lagerstedt et al. (2007) have shown small ring-shaped aggregates of apoA-IGly26Arg using transmission electron microscopy (TEM) [40]. Exhaustively exploring the grids of samples prepared for TEM, we barely detected the presence of other types of aggregates, such as disorganized protofibers in each sample (insets in Histogram images in Figure 3). However, the predominant species in our conditions, as revealed by both AFM and TEM, are oligomers. It is possible that the use of longer incubation times (up to one week) and higher protein concentrations (2 mg/mL) may have favored the formation and deposition of the larger aggregates detected in Lagerstedt’s study.

### Influence of Pro-inflammatory Conditions on apoA-I Folding and Function

**Figure 4:**
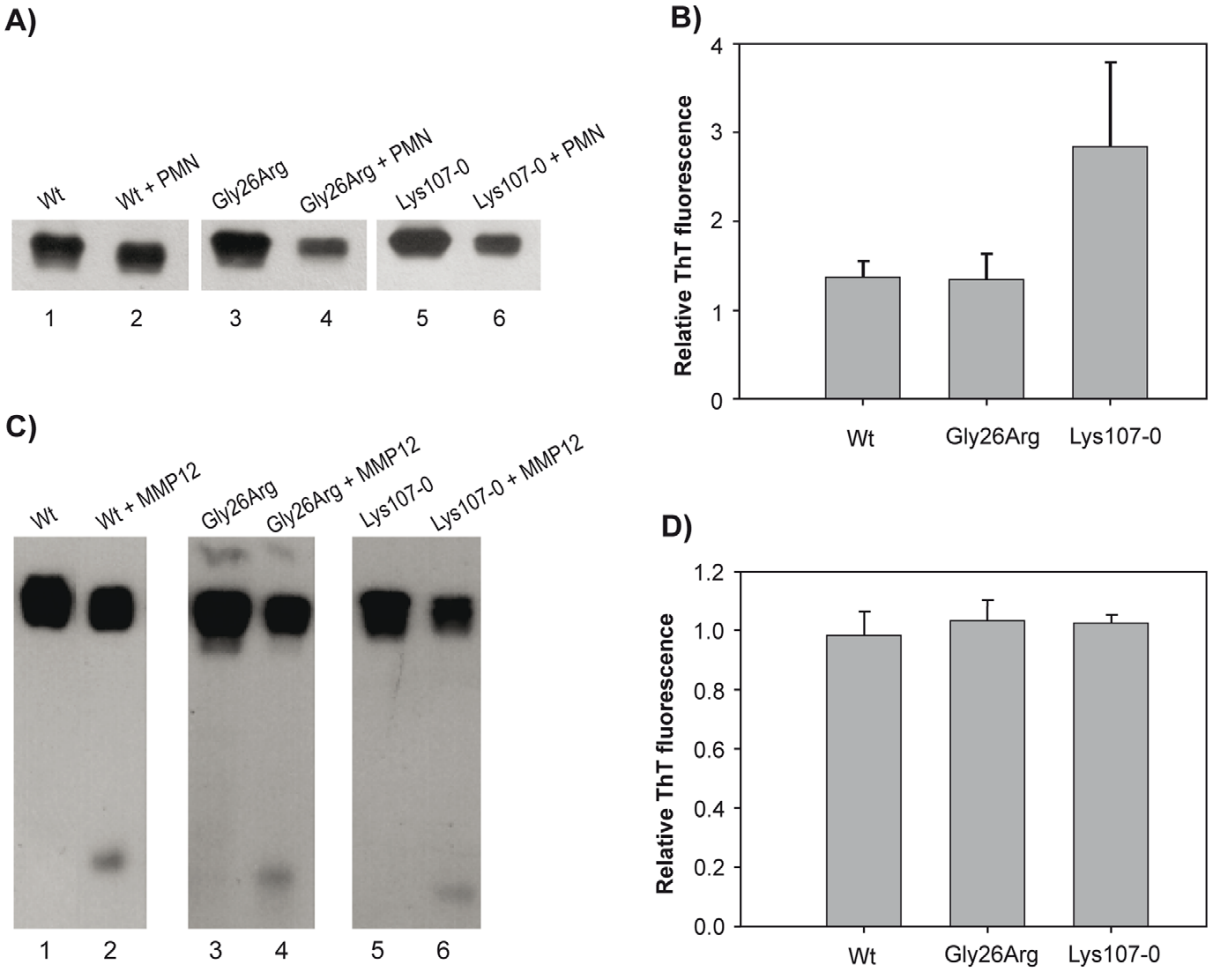
Effect of TPA-activated neutrophils and MMP-12 on apoA-I variants processing and amyloidogenicity. ApoA-I mutants (0.2 mg/mL) were incubated in the presence of TPA stimulated neutrophils at 37°C for 1 h. A) Western blotting of aliquots of the supernatant developed with rabbit polyclonal anti-apoA-I. Lanes 1, 3 and 5 show samples incubated in the absence of neutrophils, while lanes 2, 4 and 6 represent the same amount of protein applied to each lane after neutrophil treatment. B) Following the treatment with or without neutrophils samples were incubated at 37°C for 24 h. One µl of ThT (1 mM) was added to each well and ThT fluorescence was measured in a microplate reader at 480 nm (excitation at 430 nm). Each bar shows the ratio of the ThT fluorescence binding to each protein variant incubated in the presence versus the absence of activated PMNs and corresponds to means ± SE. In a different experiment apoA-I was incubated in the presence of MMP-12. C) Western blotting was developed as in A). Numbers at the bottom of each lane represent the same as in A) but in the absence or presence of MMP-12. D) After 3 h at 37°C, MMP-12 was inhibited and apoA-I incubated as in B) to determine ThT binding.

We have previously shown that incubation of Wt apoA-I with activated PMNs, associated with inflammatory response, resulted in partial protein degradation and formation of pro-amyloidogenic proteolytic products as determined by ThT binding [8]. We have now compared the behavior of apoA-I variants with that of Wt protein under the same conditions. Wt or variant forms of apoA-I were exposed to activated PMNs for 1 h (see “Methods"). As expected, proteins were partially degraded by exposure to PMNs, resulting in proteolytic products (Fig. 4A). In some experiments, degradation was considerably more drastic, with complete disappearance of the band corresponding to the intact proteins and appearance of high-molecular weight cross-linked products (not shown). Following, we analyzed ThT binding of these products. The fluorescence quantum yield of ThT is very low in aqueous buffer and is markedly increased upon binding to protein amyloids [41]–[43]. In order to better compare the different apoA-I variants, we expressed the results as the ratio of ThT fluorescence of the PMN-treated protein versus the same protein incubated in the absence of PMNs. Interestingly, while the Gly26Arg variant behaved similar to Wt apoA-I, significantly higher relative ThT binding products were obtained from the Lys107-0 variant (Fig. 4B).

A hallmark of hereditary apoA-I-induced amyloidosis is the detection of N-terminal fragments of the protein in the amyloid deposits [44]. As metalloproteinases are usually highly active at atherosclerosis plaques [45], we checked the possibility that natural mutations in apoA-I could result in the generation of a recognition site for such enzymes, and the further release of peptides with higher propensity to aggregate. To test this, we incubated proteins with metalloproteinase 12 (MMP-12) and analyzed the amyloidogenicity of the products. Interestingly, both Wt and variant forms of apoA-I were degraded by MMP-12 to similar extents, and a fragment of ∼11 kDa molecular mass could be detected (Fig. 4C). To determine whether this fragment was more prone to amyloid aggregation, we analyzed its ThT binding as described above. As observed for Wt apoA-I [8], similar ThT fluorescence intensities were measured for each apoA-I variant before and after MMP-12 proteolysis, indicating that this treatment did not significantly increase the tendency of each variant to form amyloid aggregates (Fig. 4D).

### Macrophage Activation Induced by apoA-I Variants

**Figure 5:**
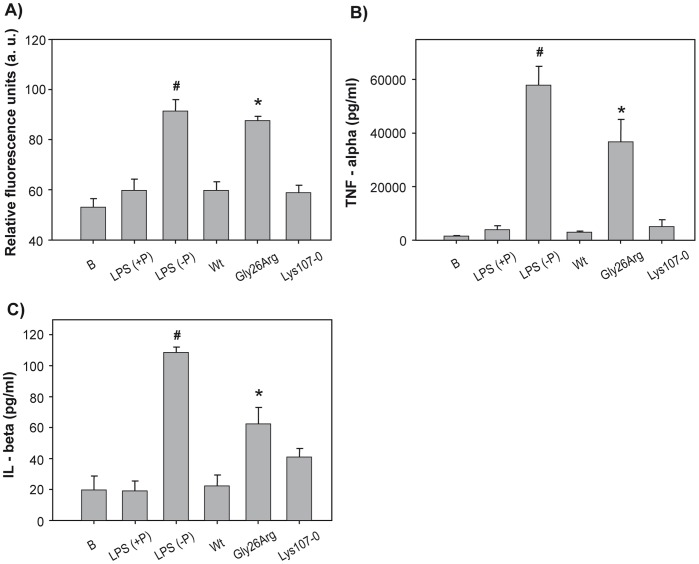
Effect of apoA-I variants in the release of pro-inflammatory species from macrophages. One μg/mL of Wt, Gly26Arg or Lys107-0 were added to RAW 264.7 macrophages previously incubated for 1 h with 50 µg/mL of polymyxin B. Lane labeled as B, as a negative control, represents the medium untreated with proteins. LPS, as a test of positive response, represents cells treated with 1 µg/mL LPS, plus (+P) or without (-P) the addition of polymyxin B. A) ROS production was measured after 6 h incubation and cell lysis by fluorescence detection of DCF formation in a microplate reader (excitation and emission filters centered at 485 and 525 nm, respectively). TNF-α (B) and IL-β (C) production is quantified as described in Methods. Bars correspond to means ± SE. Symbol # represents significant difference respect to control (in the absence of LPS) at p<0.05. * represents significant difference respect to cells incubated with the same amount of Wt protein at p<0.05.

To determine whether disease-associated apoA-I variants could induce macrophage activation, we tested their ability to stimulate production of reactive oxygen species (ROS), TNF-α and IL-1β by RAW 264.7 murine macrophages. We routinely used polymyxin B in our experiments to avoid any undesired effect of possible contaminating endotoxin in the solutions of recombinant proteins. When cells were incubated with 50 µg/mL polymyxin B, LPS-induced ROS production was completed inhibited (Fig. 5A), while, as expected, significant ROS production was detected when cells were incubated with LPS in the absence of the antibiotic. Interestingly, while neither Wt apoA-I nor the Lys107-0 variant induced ROS production by macrophages, incubation of 1 µg/mL of the Gly26Arg variant induced significant cell activation. Similarly, macrophages incubated with the Gly26Arg variant (but not with Wt apoA-I or the Lys107-0 variant) exhibited significant increases in TNF-α and IL-1β production and release (Fig. 5B, C). MTT reduction measurements indicated that cell viability was preserved under the different conditions tested (data no shown).

## Discussion

The potential to interact with a wide variety of targets constitutes the hallmark of proteins showing unstructured functional conformations. The flexible conformation required to fulfill biological functions represents, however, a potential risk of self-aggregating unfolded states. Amyloidoses are heterogeneous diseases induced by protein misfolding, in which not only protein chemical natures and propensities to self-aggregate are very different, but in addition the local environment that triggers their cytotoxicity is many times difficult to predict. We have previously proposed that wild type human apoA-I is susceptible to become pro-amyloidogenic under a local environment surrounding chronic inflammation [8]. Nevertheless, the fact that natural apoA-I variants induce amyloidosis in different organs and with different severities suggests the occurrence of other events that shift the pattern of weaker bonding modifying not only protein solubility but also inter or intra molecular interactions. Along this line, it was shown that single point mutations in transthyretin could alter both protein binding to its natural ligand T4 and the stability of the oligomeric conformation [4]. In order to get insight into the structural features that favor apoA-I amyloidogenicity, we compared the folding of the Gly26Arg and Lys107-0 variants with the folding of the Wt protein. We have worked at pH 7.4 and at low protein concentrations in order to better approximate physiological conditions. Both variants showed different conformations in the native state with respect to the Wt protein: they are less stable, showing a conformational arrangement that binds more bis-ANS and having their Trp residues more separated from each other and more exposed to the aqueous solvent than Wt apoA-I. These data suggest a more flexible structure, with increased water penetration that weakens hydrophobic interactions inducing hydrophobic domains to become exposed. Interestingly, we have noted that, when Wt apoA-I is incubated at lower pH, the bis-ANS binding sites decrease [8] and this effect is also valid for the tested mutants (not shown). As, in our hands, the secondary structures of Wt and variant forms of apoA-I are preserved during the incubation times in which experiments are performed (not shown), this suggests that, at physiological pH and after 24 h incubation at 37°C at low concentration, the mutants show a molten globule-like structure, more flexible than the Wt protein. The comparison between the unfolding profiles revealed by bis-ANS and intrinsic fluorescence measurements (Fig 2C) indicates the presence of unfolding intermediate states. These partially folded conformers can acquire an alternative and relatively stable “misfolded state," which is prone to aggregation. Partially folded intermediates have been implicated in the amyloid formation by other proteins [31], [46]–[49].

The presence of an N-terminal fragment of the protein in amyloid deposits of most apoA-I variants suggests a proteolytic processing of the precursor proteins by specific proteases, leading to formation of a peptide with higher tendency to aggregate. Indeed, a higher susceptibility of the N-terminus of apoA-IGly26Arg to chymotrypsin and V8 proteases has been reported [40]. In order to determine the influence of a pro-inflammatory environment on apoA-I aggregation, we compared the different variants as treated with MMP-12, a metalloproteinase that is highly active in inflammation. Although proteolysis was detected, this treatment did not yield a particularly pro-amyloidogenic product. Thus, although proteolytic processing is likely to occur *in vivo,* the specific enzyme involved in this event needs to be further investigated.

In addition to the pathological aggregation of the mutants, the loss-of-function of apoA-I caused by a particular mutation should also be considered. Gonzalez et al. (2008) have demonstrated that apoA-ILys107-0 looses its ability to induce intracellular cholesterol mobilization from Chinese hamster ovary cells and exhibits impaired esterification of intracellular pools [26]. The deletion of residue Lys 107 changes by about 100° the helix 4 registry and the orientation of the hydrophilic and hydrophobic faces of this amphipathic helix at both sides of the deletion point. Thus, it is possible that deletion of this residue disrupts protein conformation in a way that affects the interaction with lipids as well as with cell membrane proteins involved in triggering the signaling pathways leading to the mobilization of intracellular cholesterol pools.

As apoA-ILys107-0–derived amyloid occurs associated to severe atherosclerotic plaques in the intima [50] a close connection between these two chronic diseases could be predicted. Our results demonstrated a clear tendency of this mutant to aggregate even at low protein concentration and, in addition, a higher sensitivity to yield pro-amyloidogenic products by inflammation-induced oxidation/proteolysis. Another well-recognized general mechanism of *in vivo* amyloidogenesis is the generation of a critical concentration of the amyloidogenic precursor [7]. Thus, it is possible that the decreased efficiency of apoA-ILys107-0 to acquire lipids, together with a local concentration of macrophages in the atherosclerotic lesion, could render a higher amount of lipid-free protein [9] that, due to its inherent tendency to misfold, could deposit in the intima in close association to the atherosclerotic plaque. In this regard, the concentration of lipid-free apoA-I in the aortic intima has been shown to increase during the progression of atherosclerosis [51].

Interestingly, in spite of the lower levels in total plasma HDL and apoA-I, no relevant atherosclerosis or cardiovascular disease have been reported for apoA-IGly26Arg carrying patients. Instead, the Gly26Arg variant has been associated to renal disease, polyneuropathy [52], [53] and hepatic dysfunction [54]. It has been suggested that introduction of a positive charge by this mutation induces repulsive interactions with Lys23, increasing solvent exposure of this region of apoA-I [55]. In spite of the observed structural differences, destabilization is necessary but probably not sufficient to confer an amyloidogenic propensity on a protein [56]. As shown here, despite being less stable, under our mild incubation conditions apoA-IGly26Arg does not show a higher tendency to aggregate or been processed by neutrophils than Wt apoA-I.

In addition to a higher rate of catabolism, it has been suggested that lower plasma levels of apoA-I are due to the fact that protein is sequestered in extravascular tissues [24]. Any local factors that perturb the three dimensional structure of the protein, such as pH, temperature, osmolytes, or urea in the inner renal medulla could enhance the formation of partially folded states that either deposit or increase the retention time in the capillaries [56]. Binding to plasma membranes enriched in negative phospholipids, such as those of apoptotic cells, or to components of the extracellular matrix, as the glomerullar basal membrane in the kidneys, could be increased by the presence of an extra positive charge in the N-terminus. Along this line, we have suggested that protonation of histidine residues in Wt apoA-I results in formation of a heparin binding site [8]. Impaired interactions with less-known partners could be also envisaged, as it was proposed that the N-terminal domain of apoA-I is involved in the *in vitro* blockade of the neurotoxicity of the Aβ peptide [57].

An additional mechanism that should be considered in order to understand a hallmark of apoA-I-associated amyloidosis is that misfolded proteins could induce activation of cellular responses that trigger chronic inflammation in an attempt to clear up the anomalous protein conformer. Indeed, it has been shown that extracellular aggregated human α-synuclein activates microglia, inducing the release of pro-inflammatory mediators that elicit the progression of Parkinson disease [58]. In order to examine this possibility, we investigated the effects of the apoA-I variants on RAW 264.7 murine macrophages. Interestingly, under our conditions apoA-IGly26Arg, but not Wt apoA-I or apoA-ILys107-0, induced ROS, TNF-α and IL-1β production by the macrophages, raising the possibility that the subtle changes in protein conformation and/or the change in amino acid sequence of the Gly26Arg variant could initiate and/or perpetuate a local inflammatory condition associated to organ dysfunction.

In conclusion, we have analyzed and compared structural features that could favor amyloid aggregation of two known natural variants of apoA-I. Some structural features appear to be shared by both variants, such as the presence of a more flexible and unstable structure than in the Wt protein. Nevertheless, this fact do not seem to be sufficient to explain the increased pathogenicity they show with respect to Wt apoA-I, as the Gly26Arg variant behaves similar to Wt as far as yielding amyloid aggregates, indicating that other local factors likely determine the shift in equilibrium between fully folded and partially folded pro-amyloidogenic forms. In addition to the effect that the local microenvironment could exert on apoA-I conformation, it should be considered that misfolded proteins could indeed mediate other cell signaling events, determining an intricate cross-talk between function and pathogenicity. Induction of macrophage activation could be an attractive hypothesis to explain why certain variants contribute more than others to apoA-I-induced pathogenesis. Further studies should focus on the complex landscape mediating the roles of apoA-I in the delicate balance between health and pathology.
